# Extinction, Slime, and Bottoms

**DOI:** 10.1371/journal.pbio.0020272

**Published:** 2004-08-17

**Authors:** Sean Nee

## Abstract

Is biodiversity decreasing or is it being replaced by a splendid profusion of microbes -- and does this matter?

There is an old Chinese curse: ‘May you live in interesting times.’ According to those who know about such things, we live in a momentous time, the time of the Sixth Mass Extinction! But most of us do not feel at all cursed. Because, in fact, the Sixth is quite different to the previous Big Five—no-one would notice this one if we were not repeatedly reminded of it by ecologists. Previous mass extinctions were not so bashful, so discreet. The fossil record reveals the disappearance of pollen during previous ones, replaced by an abundance of fungus spores, telling us of a world of devastated forests rotting away. The earliest sediments after the mass extinction that did away with the dinosaurs are barren of fossils: so it is not just that species were going extinct, conditions for life itself were bad. Not only did species diversity drop, the abundance of life did as well.

But conditions for life itself have never been better than today. In the history of the planet, there has never been anything as productive of life as a wheat field in Kansas. It may not have a large diversity of species, but that is a different matter. In fact, one of the reasons for the ongoing loss of plant diversity from grasslands is the very reason the wheat field is so productive—fertilisation. We are pouring nitrogen fertiliser into the environment and, through the wellstudied ‘paradox of enrichment’, this reduces species diversity while increasing actual biomass. Now, there is no question that if current trends of habitat alteration and climate change continue then we will ultimately lose large numbers of species—diversity will drop—but this does not necessarily translate into a loss of abundance of life, and that is a big difference between now and previous mass extinctions. Looking at specific groups of organisms tells the same story. So, for example, many island bird species are threatened, like the kagu of New Caledonia, but British seabird populations, like puffins, are booming. Worldwide amphibian diversity is threatened, but cane toads are a pest in Australia. Introduced species pose a threat to diversity—the ‘McDonald-isation’ of nature—precisely because they achieve enormous abundances.

Actually, all six mass extinctions may have one very important thing in common: from the point of view of the vast bulk of life on the planet they are probably not mass extinctions at all. By any criterion—number of individuals or total biomass—the vast majority of life on earth is invisible—microbial. So, for example, at least 10% of the living biomass on earth consists of bacteria living deep in the oceans' sediments: it would take more than an asteroid impact to disturb them. And microbial life is extraordinarily robust: microbes can be found living happily in pressurised water hotter than your boiling kettle, in concentrated acid, and in rock, and their spores can survive for years in the rigours of outer space.

In talks and lectures, the renowned oceanographer and paleontologist Jeremy Jackson paints a vivid picture of what is currently happening to coastal ecosystems, talking about a wall of slime emanating from populated areas and growing outwards inexorably towards the open oceans, replacing beloved ecologies like coral reef systems. What he means is that the visible life that we find attractive and useful—pretty fish, turtles, and so on—is being replaced by microbes in splendid profusion. It is taken completely for granted that this is disastrous. From a utilitarian point of view indeed it is disastrous, since we like eating fish and turtles, and don't like snorkling in slime. But from the point of view of life per se, again things have never been better. Life is so abundant that in some places all the oxygen in the water is completely used up. These are called ‘dead zones’, but they are no more ‘dead’ than the Dead Sea, which is actually teeming with life—just not fish. But, nonetheless, we consider what is occurring to be a disaster not just from a utilitarian point of view, but at some deeper level giving us an emotional reaction to the word ‘slime’—somehow it is just plain wrong.

But this reflects nothing other than our evolutionary origins. Evolution has programmed us to be positively interested in plants and animals, our food, and to be repelled by slimes and oozes, teeming with potentially harmful microbes. These emotional responses colour our view of ecology, for example, in a way that has no parallel in other sciences: physicists do not just study particles that they find pretty. No ecologist wants to study the rich ecosystem that each of us carries around inside our gut, because evolution has programmed our brains to find bottom-related matters disgusting. I think it likely that naturalists from a different planet, silicon entities evolved under very different circumstances, would find tropical forests uninteresting (mainly primary producers with some herbivory and mutualisms) and animal guts fascinating, with their complex metabolic networks in which each node is manned by different species with wildly varying means of energy production.

Our guts should be an ecological scientist's dream come true, ecological theatres that are replicated billions of times, which operate on a fast time scale and are easy to get to! (If aliens are ecologists, this would explain why they always ‘probe’ their abductees.) Many natural experiments are going on all the time as antibiotics and probiotics are administered and people find all sorts of different ways, voluntary or otherwise, to establish migration links between their gut ecologies. Microbiologists are increasingly interested in our guts from an ecological point of view but, unlike ecologists, they are used to faeces from their work in sewage plants.

Two thousand years ago the Roman senator Cicero noted the creation of barren desert-like land in North Africa after the forests were felled for their timber, providing the earliest record of an ecosystem ‘service’ provided by forests—the stabilisation of soils. Other services provided by biodiversity readily come to mind, like pollination and carrion clean-up, and there may be many more. But perhaps the clearest example of an ecosystem service provided by biodiversity comes from our gut. Throughout our history, until very recently, we all had worms. In rich countries we have quite happily eradicated them from our inner ecosystems with none of the handwringing we expend on rhinos. But it is increasingly believed that the loss of worms from our internal ecology is responsible for the upsurge in inflammatory bowel disorders such as Crohn's disease and colitis. In fact, there are clinical trials underway in the United States testing the efficacy of worms as treatment for these diseases. The mechanism is clear: worms trigger one arm of the immune system which down-regulates another, inflammatory arm. Our immune system has evolved to expect a certain constellation of species in our gut: in that context, worms provide an ecosystem service of balancing the immune system.

Our perception of our impact on the planet as equivalent to a mass extinction simply reflects the evolutionary prism through which we view life. Of course, we may yet live up to our own publicity and pull off something apocalyptic like a runaway greenhouse that sterilises the Earth. But it is at least as likely that the microbial world, resentful at being either ignored or exterminated, will come up with something to consign us to a footnote in the history of life when it is ultimately written by the silicon entities. The Spanish flu, SARS, and HIV have just been early experiments.

